# Bristol (Mass.) South District Medical Society

**Published:** 1855-12

**Authors:** 


### *Bristol (Mass.) South District Medical Society.*

Messrs. Editors: Being in New Bedford on Wednesday, 14th inst., I was politely invited to attend the semi-annual meeting of the "Bristol South District Medical Society," then holden in the City Hall. A goodly number of the members were present, and activity, with great harmony, prevailed. After the ordinary business was over, a very interesting and remarkably well written paper, on some of the causes of the prevalent or so-called "fashionable" diseases of females, was read by one of the members, Dr. Comstock, of Middleborough. His position appeared to be just and reasonable—founded on accurate observation and good common sense. Among other things he instanced the present manner of suspending so great an amount of clothing, tightly girt, from the hips; and defended the superiority of the old-fashioned stays—an opinion in which most unprejudiced observers will probably agree. He further seemed to think that much more was to be gained by a discriminating general treatment than by local applications. And from subsequent remarks from other members it might be inferred that the *specialty* was not in very good repute in Bristol South. The Society may be congratulated in having the subject so judiciously discoursed upon.

After Dr. Comstock's paper, an extempore discussion arose on a question incidentally started by one of the members, in which most of those present took part. The discussion was animated, gentlemanly, and dignified. Our fraternity are too apt to discuss the expediency of giving more or stronger medicines, but in this case, the *safety of omitting* a certain drug in the disease in question, was debated—a novel but very gratifying innovation.

The relation of several cases followed. At half past one o'clock, the Society adjourned to the Parker House, to the dinner given by the physicians of New Bedford.

The Bristol Society have every reason to be satisfied with the meeting on Wednesday; and for their hospitalities on that occasion they have the grateful acknowledgments of at least one from—*Ib.* NORFOLK.

